# CRAWLING: a crowdsourcing algorithm on wheels for smart parking

**DOI:** 10.1038/s41598-023-41254-7

**Published:** 2023-10-03

**Authors:** Émiland Garrabé, Giovanni Russo

**Affiliations:** Department of Information and Electrical Engineering and Applied Mathematics, University of Salerno, 84084 Fisciano, Italy

**Keywords:** Electrical and electronic engineering, Applied mathematics

## Abstract

We present the principled design of CRAWLING: a CRowdsourcing Algorithm on WheeLs for smart parkING. CRAWLING is an in-car service for the routing of connected cars. Specifically, cars equipped with our service are able to *crowdsource* data from third-parties, including other cars, pedestrians, smart sensors and social media, in order to fulfill a given routing task. CRAWLING relies on a solid control-theoretical formulation and the routes it computes are the solution of an optimal data-driven control problem where cars maximize a reward capturing environmental conditions while tracking some desired behavior. A key feature of our service is that it allows to consider stochastic behaviors, while taking into account streams of heterogeneous data. We propose a stand-alone, general-purpose, architecture of CRAWLING and we show its effectiveness on a set of scenarios aimed at illustrating all the key features of our service. Simulations show that, when cars are equipped with CRAWLING, the service effectively orchestrates the vehicles, making them able to react online to road conditions, minimizing their cost functions. The architecture implementing our service is openly available and modular with the supporting code enabling researchers to build on CRAWLING and to replicate the numerical results.

## Introduction

By 2050, it is expected^1^ that 66% of the World population will live in urban environments, with many of our cities set to become *megacities*. Hence, also driven by the *Internet of Things* (IoT) revolution and the explosion in the amount of available data, many governments and local authorities have adopted (or are in the process of adopting) smart cities concepts to tackle the key challenges related to the growing intensive urbanization^2^. As a result, much research and development effort is currently devoted to the design of IoT and data-driven services with the goal of making cities sustainable^3^, capable of improving the quality of life of their Citizens with subsequent societal, economic and environmental impacts. The design of advanced transportation systems is central to achieve each of these goals and an exciting opportunity is offered by the possibility of designing, and deploying, services leveraging the increasing pervasiveness of *connected cars*^4^, i.e., vehicles capable of accessing the Internet, communicating with other devices and cars through vehicle to vehicle and/or vehicle to infrastructure communication. In this context, we propose the principled design of a data-driven service based on crowdsourcing for the routing of connected cars, CRAWLING (CRowdsourcing Algorithm on WheeLs for smart parkING). Connected cars equipped with CRAWLING are able to crowdsource the skills/services they need in order to fulfill a given routing task. The service is also designed to allow cars equipped with CRAWLING to gather data from other sources, such as other cars, pedestrians, smart sensors or social media. As a result, vehicles equipped with CRAWLING become an active part of a sharing economy^5^ ecosystem, where vehicles share, and create, *skills* that can be used within the service. A key feature of our service is that it is built around a framework that intrinsically allows to consider stochastic behaviors, thus explicitly accounting for users’ stochasticity and to e.g., capture their privacy requirements. Specifically, CRAWLING exploits a recent data-driven control algorithm^6,7^ that returns a randomized behavior for the car (i.e., a probability function) by solving a sequential decision-making problem^8^ that formalizes the tracking of a desired behavior expressed in probabilistic terms. That is, our service systematically minimizes a cost function consisting of: (i) a reward capturing road/traffic conditions; (ii) a regularizer that biases the solution towards some target behavior, allowing CRAWLING to keep into account the possible preferences of passengers.

**Related works.** While CRAWLING can be tailored towards generic routing problems, for concreteness we focus here on the problem of routing cars to find parking spaces. Smart parking allocation is attracting much research attention^9–12^, as roaming for parking is known to be a major source of pollution, traffic and user stress^13–15^. In a broader context, lack of coordination in transportation systems is believed to be correlated to an increase in circulating vehicles^16^ and reducing parking-related traffic is a question of interest in this context^17^. To mitigate this, in the context of parking management, urban sensors can be used to monitor the availability of parking spaces^18,19^, with proposals to use sensors to automatically detect parking availability or provide automated booking/charging services^20^. Efforts to leverage users’ data in the broad context of smart cities are also the focus of much attention^21,22^, for example for energy^23^ or taxi^24^ demand prediction, or congestion analysis^25^. For parking applications, a key challenge becomes that of designing algorithms that are able to optimally route the flow of vehicles^26^ by leveraging these data. Currently, many such algorithms rely on deterministic decision-making^27^. For example, methods^28^ for solving multi-depot vehicle routing frame the problem via deterministic optimization, using binary decision variables. On the other hand, the use of stochastic frameworks has been gaining traction in the literature on advanced and intelligent transportation systems, for example in the context of differential privacy (privacy is indeed a key requirement for these applications^29^) where privacy is achieved by injecting noise in the system^30,31^, or in driver^32^ or pedestrian^33^ intent prediction. Probabilistic methods/models^34^ have a long history^35^ in mobility estimation/prediction. Among other features, these models can account for users’ stochasticity and uncertain, non-optimal, behaviors: we recall here probabilistic models for human mobility^36^, stochastic filters for vehicle behavior estimation and prediction^37^ or behavioral models inferred through learning^38^. Efforts have also been made to derive probabilistic traffic models, for example using Bayesian networks^39^ or physically-inspired flow models based on PDEs^40^. While such efforts often focus on modelling^41^ and/or artificial intelligence methods^42,43^, some specialized stochastic optimization frameworks have also arisen, for example in parking assignment^44^. Tools from stochastic optimal control can be leveraged to manage parking. Stochastic parking assignment has been shown to have the potential to outperform deterministic methods^45^. In relation to this, some works envision stochastic energy pricing strategies for parked electrical vehicles^46^, or controlling parking occupancy through entry prices determined by solving a stochastic optimization problem^47^. Also for this stream of literature, the design of services that can run in real time^48^ and the orchestration of heterogeneous data have been identified as key challenges^49^. In this context, decision engines^50^ have been recently designed that enable agents to make decisions by merging streams of available data. The engines, which leverage the probabilistic framework^8^ also used in this paper, have been also applied to parking management. Finally, data-driven intelligent transportation systems, and their data-driven analysis^51^ have also gained traction, although heterogeneous datasets, emerging from different information streams that need to be merged, have been singled out as a challenge^52,53^. As an example of one such stream, social media monitoring has been recently used to identify trends related to traffic obstructions^54^ and monitor traffic in real time^55^.

The key contributions of the paper can be summarized as follows: after introducing the smart parking problem as a data-driven control problem, we propose the principled design of CRAWLING and present a stand-alone, general-purpose, modular architecture of the service that is made openly available https://tinyurl.com/wcwvcwaj. The architecture we propose is built to gather online data from the environment, from other cars equipped with CRAWLING and from certain social media. Moreover, the service provably returns the optimal behavior for cars on which it is equipped. Then, we evaluate its computation times and discuss how these are suitable for routing applications. We also show, via simulations leveraging the microscopic traffic simulator SUMO^56^, the effectiveness of CRAWLING in orchestrating the use of parking spaces in both a University Campus under different operating conditions and in a larger city center scenario. Simulations show that CRAWLING effectively directs the connected cars, confirming that vehicles equipped with our service improve their average time-to-parking, avoiding unfavourable areas of the map and reacting online to environmental changes (that can be reported, as in one of the scenarios, via social media). Our supporting code is openly available, providing two versions of our architecture (differing in the way the service interacts with social media) which is built to be modular. This facilitates researchers to build on CRAWLING and allows users to replace some of the components of our service so that performance of its different parts can be assessed and benchmarked. For example, the architecture we make available allows to embed different decision engines in CRAWLING, such as recent decision-making algorithms^50^ for data composition. In this context, we highlight that the social media dimension, the CRAWLING architecture and its validations (including a city-wide scenario) are specific contributions of this paper.

The paper is organized as follows. In “Materials and methods”, we begin by introducing the data-driven control framework exploited by CRAWLING, and subsequently describing its design, the architecture and the scenarios used to assess its performance. Then, in “Results”, we report the results obtained with the service. We do so by first evaluating the computational efficiency and then discussing the performance obtained on the reference scenarios, which were specifically chosen to validate all the key features of the service. Finally, we give concluding remarks, and discuss the relevance of the results, Section 4.

**Table 1:** Notation table.

Symbol	Description
$\mathscr {X}$	Set of road links/lanes
*k*	As a subscript, time index
*N*	Width of time horizon for Problem 1
$\textbf{x}_{k-1}$	Road link occupied by the car (state)
$r_k(\textbf{x}_k)$	Reward associated to a road link at time step *k*
$\tilde{r}_k(\textbf{x}_{k-1})$	Expected reward, $\mathbb {E}_{\pi (\textbf{x}_k\mid \textbf{x}_{k-1})}\left[ r_k(\textbf{X}_k)\right]$
$\pi (\textbf{x}_k|\textbf{x}_{k-1})$	Car turning probability
$p(\textbf{x}_k|\textbf{x}_{k-1})$	Target turning probability
$\pi (\textbf{x}_1,\ldots ,\textbf{x}_N|\textbf{x}_0)$ and $p(\textbf{x}_1,\ldots ,\textbf{x}_N|\textbf{x}_0)$	Car and target behaviors from $\textbf{x}_0$
$p(\textbf{x})$	Probability Mass Function (pmf)
$\mathbb {E}_p\left[ \cdot \right]$	Expectation symbol
$\mathscr {D}_{KL}\left( \cdot \mid \mid \cdot \right)$	KL divergence (arguments are pmfs)
$\{w_k\}_{k_1:k_2}$	The set $\{w_{k_1},\ldots ,w_{k_2}\}$
$\{\pi ^{(i)}(\textbf{x}_k|\textbf{x}_{k-1})\}_{1:N}$	Sequence of turning probabilities from the *i*-th source
$\varvec{\alpha }_k$	*S*-dimensional weight vector used (Problem 1) to combine the sources
$p(\textbf{x}_k|\textbf{x}_{k-1})$	Target behavior at time step *k*
$\pi (\textbf{x}_k|\textbf{x}_{k-1})$	Car transition probability (behavior) at time step *k*
$\tilde{\pi }(\textbf{x}_k|\textbf{x}_{k-1})$	Optimal car turning probability at time step *k* (determined from Algorithm 1)

### Notation

We note the set of road links, or lanes in what follows, as $\mathscr {X}$. Time is discrete and, at time step *k*, the connected car occupies link $\textbf{x}_k\in \mathscr {X}$. The probability of a connected car of going from $\textbf{x}_{k-1}$ to $\textbf{x}_k$, i.e., the car turning probability, is denoted by $\pi (\textbf{x}_k|\textbf{x}_{k-1})$. The car has acces to a number of services (or sources) and the *i*-th service/source provides a turning probability denoted by $\pi ^{(i)}(\textbf{x}_k|\textbf{x}_{k-1})$. The target behavior of the car represents the behavior that passengers would like to track in the absence of road (or environmental) disruptions: this is denoted by $p(\textbf{x}_{k}|\textbf{x}_{k-1})$. Finally, the reward obtained by the car at time *k* for being on $\textbf{x}_{k}$ is denoted by $r_k(\textbf{x}_k)$. As we shall see, the reward is used in our service to capture road/environmental disruptions that might not be known to the vehicle passengers. Also, we denote by $\pi (\textbf{x}_1,\ldots ,\textbf{x}_N|\textbf{x}_0)$ and $p(\textbf{x}_1,\ldots ,\textbf{x}_N|\textbf{x}_0)$ the product of the transition probabilities $\pi (\textbf{x}_k|\textbf{x}_{k-1})$ and $p(\textbf{x}_{k}|\textbf{x}_{k-1})$, respectively. These joint probability functions clearly depend on the initial condition of the car, $\textbf{x}_0$, i.e. the starting link/lane of the trip. Also, we let $\mathbb {E}_p[h(\textbf{X})]:= \sum h(\textbf{x})p(\textbf{x})$ be the expectation of $h(\cdot )$ with the sum taken over the support of the probability function $p(\textbf{x})$. The Kullback-Leibler (KL) divergence^57^ of $p(\textbf{x})$ with respect to $q(\textbf{x})$, with $p(\textbf{x})$ absolutely continuous with respect to $q(\textbf{x})$ is $\mathscr {D}_{\text {KL}}\left( p(\textbf{x}) \mid \mid q(\textbf{x}) \right) := \sum p(\textbf{x}) \log \frac{p(\textbf{x}) }{q(\textbf{x}) }$ and measures the proximity of the pair of pmfs $p(\textbf{x})$, $q(\textbf{x})$. Specifically, the KL divergence is non-negative and zero if and only if $p(\textbf{x})=q(\textbf{x})$. We use the notation $k_1:k_2$ to denote the set of integers between the integers $k_1$ and $k_2$ (included). Consequently, $\{w_k\}_{k_1:k_2}$ denotes the set $\{w_{k_1},\ldots ,w_{k_2}\}$. For a comprehensive summary of notation/symbols used throughout the paper refer to Table 1.

**Figure 1:**
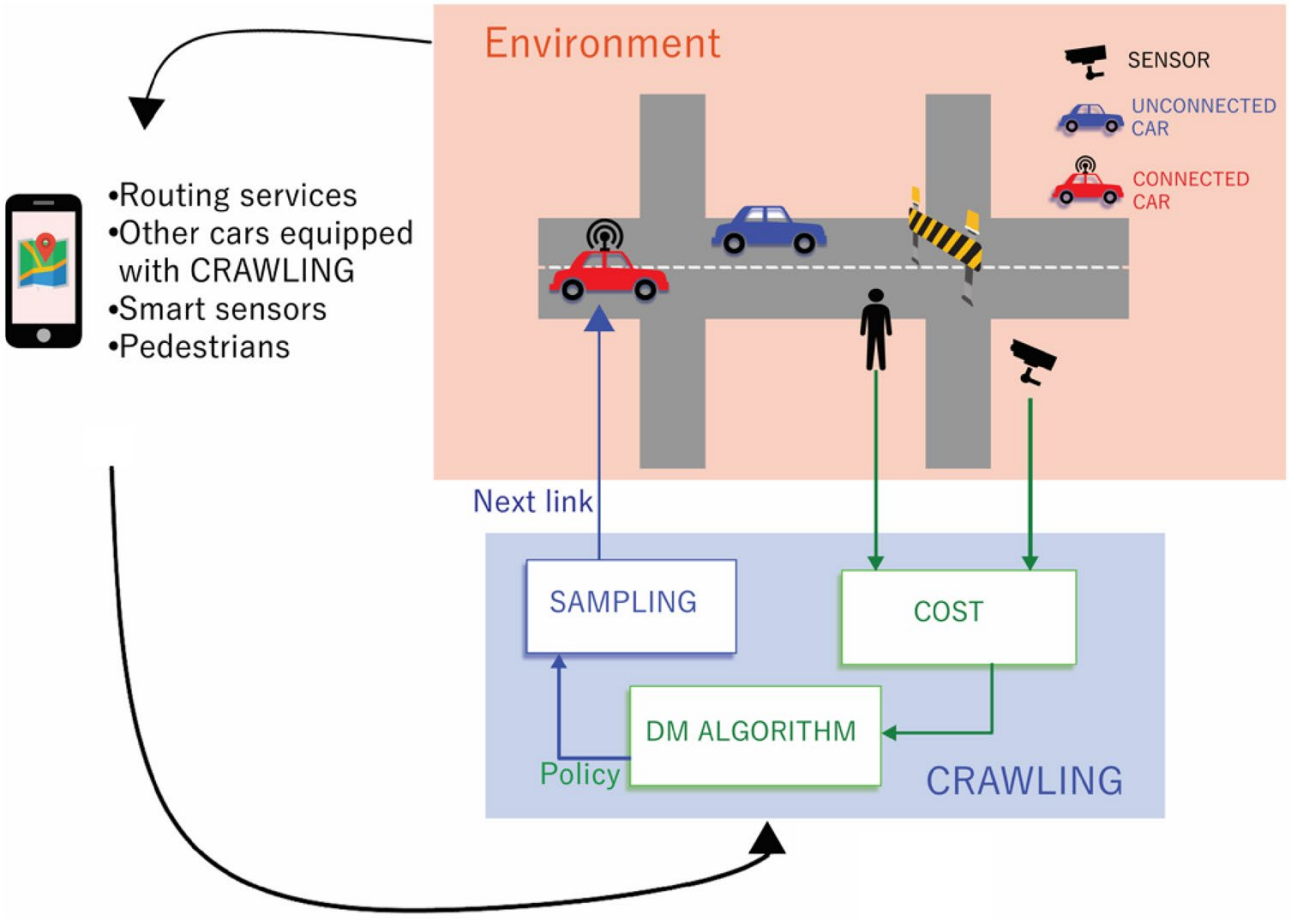
CRAWLING functional architecture and its interactions with the environment. CRAWLING is an in-car service and consists of a decision-making algorithm, a cost module and a sampling mechanism. These components compute the route the car should take based on environmental data and on the available services.

## Materials and methods

We now introduce CRAWLING. We first present the algorithm (Section "CRAWLING: description of the service") and then discuss its implementation (Section "Making CRAWLING architecture openly available: implementation and validation set-up") and the reference scenarios used to assess its performance (Section “Scenarios description”). The key functional components of CRAWLING, and its interactions with the surrounding road environment, are schematically illustrated in Fig. 1. As shown in such a figure, CRAWLING is an in-car service: it gathers online data from the environment to feed a suitably defined cost function and has a number of source services (or simply sources in what follows) to route the car. The sources are e.g., specialized routing services as well as directions collected from other cars in the area also equipped with CRAWLING. Based on the cost and the available sources, CRAWLING dynamically determines the next direction the car should take. This is done via a decision-making (DM) algorithm that exploits recent results in the context of data-driven control^6,7^. The DM algorithm returns a probability function (i.e., a policy) capturing the turning probabilities of the car. The next road link that the car should take is then obtained by sampling form the policy/turning probability.

### CRAWLING: description of the service

CRAWLING seeks to determine the optimal turning probabilities, say $\tilde{\pi }(\textbf{x}_k|\textbf{x}_{k-1})$, of the car by orchestrating the use of a set of available sources. The goal is to track a target behavior for the car, say $p(\textbf{x}_k|\textbf{x}_{k-1})$, while simultaneously maximizing the car-specific reward $r_k(\textbf{x}_k)$. The target behavior can be used to capture a preferred set of directions for the car passengers, which can be computed from e.g., historical data^58–60^. Road conditions during the trip can be captured through the car-specific reward $r_k(\textbf{x}_k)$ and, within the service, the reward signal is used to capture data that are not included in the target behavior: typically, these are related to the state of the network, including hazardous conditions, traffic and roadworks. Such data can be gathered from a network of urban sensors (Scenario 1 in Section “Scenarios description”) and the reward can be also be fed by interactions with social media (see Section "Making CRAWLING architecture openly available: implementation and validation set-up" and Scenario $1.\text {c}$ in Section “Scenarios description”). For example, in the simulations from Section “Results”, a component of the reward favours road links adjacent to an available parking space, encouraging agents to park. This structure allows the reward to encompass specifications linked to various tasks, meaning CRAWLING is not per se limited to parking management.

We now describe our service and we do so by first introducing the underlying optimal control problem, then presenting the optimal solution and finally introducing the decision-making (DM) algorithm (we refer to Table 1 for the notation). Say *S* the number of sources that are available to the car, i.e., $\pi ^{(i)}(\textbf{x}_k|\textbf{x}_{k-1})$ with $i=1,\ldots ,S$ (see Section "Notation" for the definitions). These turning probabilities are assumed to be such that $\mathscr {D}_{\text {KL}}\left( \pi ^{(i)}(\textbf{x}_k\mid \textbf{x}_{k-1})\mid \mid p(\textbf{x}_k\mid \textbf{x}_{k-1})\right) < +\infty$. This is a standing assumption for our service that has been shown to be non-restrictive in practice^6^: as we shall see, the turning probabilities from the sources are an input to the CRAWLING DM algorithm and hence, upon receiving them, the algorithm can use only the pmfs that satisfy this assumption. Given this set-up, the problem tackled by CRAWLING to determine the optimal route for the car can be formalized by leveraging the optimal control formulation^6,7^:

#### Problem 1

Let *N* be a positive integer, $\tilde{r}_k(\textbf{x}_{k-1}) := \mathbb {E}_{\pi (\textbf{x}_k\mid \textbf{x}_{k-1})}\left[ r_k(\textbf{X}_k)\right]$ and $\pi (\textbf{x}_0)$, $p(\textbf{x}_0)$ be priors on the initial conditions. Find the sequence of weights ${\left\{ \varvec{\alpha }_k^*\right\} _{1:N}}$, with $\varvec{\alpha }^{*}_k:=[\alpha _k^{(1)},\ldots ,\alpha _k^{(S)}]$ being an *S*-dimensional vector at time step *k*, solving

1 $$\begin{aligned} \underset{\left\{ \varvec{\alpha }_k\right\} _{1:N}}{\text {min}}&\mathscr {D}_{\text {KL}}\left( \pi (\textbf{x}_1,\ldots ,\textbf{x}_N|\textbf{x}_0)\pi (\textbf{x}_0)||p(\textbf{x}_1,\ldots ,\textbf{x}_N|\textbf{x}_0)p(\textbf{x}_0)\right) - \sum _{k=1}^N\mathbb {E}_{\pi (\textbf{x}_{k-1})}\left[ \tilde{r}_k(\textbf{X}_{k-1})\right] \nonumber \\ s.t.&\ \pi (\textbf{x}_{k}|\textbf{x}_{k-1}) = \sum _{i\in 1:S}\alpha _k^{(i)}\pi ^{(i)}(\textbf{x}_{k}|\textbf{x}_{k-1}), \ \ \ \forall k \nonumber \\&\sum _{i\in 1:S}\alpha _k^{(i)} = 1, \ \ \alpha _k^{(i)} \in \{0, 1\}, \ \ \forall k. \end{aligned}$$

In Problem 1 the cost function formalizes the fact that the goal of CRAWLING is to track the target behavior while maximizing the car-specific reward. The constraints capture the fact that the solution of the problem is a probability function and, in turn, this probability function is determined by picking one of the sources available to CRAWLING. At each *k*, the optimal solution to the problem can be shown^6,7^ to be of the following form:

$$\begin{aligned} \tilde{\pi }(\textbf{x}_{k}|\textbf{x}_{k-1}) = \sum _{i\in 1:S}\alpha _k^{(i)*}\pi ^{(i)}(\textbf{x}_{k}|\textbf{x}_{k-1}), \end{aligned}$$

where $\alpha _k^*$ is a weight vector having all zeros except one element, say $j_k$, equal to 1. Specifically, $j_k$ can be computed^6,7^ as

$$\begin{aligned} j_k \in \underset{i\in 1:S}{\text {arg min}}\mathscr {D}_{\text {KL}}(\pi ^{(i)}(\textbf{x}_{k}|\textbf{x}_{k-1})\mid \mid p(\textbf{x}_{k}|\textbf{x}_{k-1})) - \mathbb {E}_{\pi ^{(i)}(\textbf{x}_{k}|\textbf{x}_{k-1})}[\bar{r}_k(\textbf{X}_k)], \end{aligned}$$

where $\bar{r}_k(\cdot )$ is obtained via the backward recursion

$$\begin{aligned} \bar{r}_k(\textbf{x}_k)&:= r_k(\textbf{x}_k) + \hat{r}_k(\textbf{x}_k)\\ \hat{r}_k(\textbf{x}_k)&:=\underset{i}{\text {min}}\mathscr {D}_{\text {KL}}(\pi ^{(i)}(\textbf{x}_{k+1}|\textbf{x}_{k})\mid \mid p(\textbf{x}_{k+1}|\textbf{x}_{k})) - \mathbb {E}_{\pi ^{(i)}(\textbf{x}_{k+1}|\textbf{x}_{k})}[\bar{r}_{k+1}(\textbf{x}_{k+1})]\\ \hat{r}_N(\textbf{x}_N)&:= 0 \end{aligned}$$

We refer to prior work^6,7^ for the full derivations with a detailed statement of the technical result. The derivations, which allow to explicitly compute the optimal solution of Problem 1, can be implemented via an algorithm: this is Algorithm 1, which embeds both the *cost* and *DM algorithm* components of the service in Fig. 1. The key macro-steps of the algorithm, which outputs the optimal solution to Problem 1, are described next.

The inputs to the algorithm (lines 1–6) are, besides reward and sources, a time horizon, *N*, and the current state of the agent, $\textbf{x}_{k-1}$. The target behavior is an optional input parameter. Indeed, if not provided to CRAWLING, this is set to the uniform distribution (lines 7–9). We note that, when this happens, the first term in the cost of the problem in (1) becomes an entropic regularizer, also widely used in the literature on reinforcement learning^61^. The output of the algorithm (lines 10–11) are the optimal turning probabilities throughout the time horizon. This is essentially the plan that the car will follow. Following the initialization phase, the for loop computes the *reward-to-go* for the car (this is computed by the *cost* module in Fig. 1) which represents how *promising* a given direction plan is for the car. The reward-to-go is computed via a backward recursion that starts from the last time step in the time horizon. Intuitively, the time horizon is a measure of *how far in the future* CRAWLING can look to make a decision (see Section "Computational performance" for the corresponding computational aspect). Specifically, in Algorithm 1, the reward signal $r_k(\cdot )$ received by CRAWLING is updated with information from the future time steps through the signal $\hat{r}_k(\cdot )$, which is obtained via backward recursion. Then, the cost of each source’s policy is calculated (lines 17–18) and the source incurring the smallest cost is selected, giving the car’s turning probabilities—this is returned in line 23. Finally, the next link that the car needs to take is sampled (see the *Sampling* component illustrated in Fig. 1) from the turning probability.

Before reporting on the implementation of CRAWLING, we also highlight here a benefit in computing (and using as input) probability functions. First, the sources used by CRAWLING are intrinsically stochastic and this can be useful when the agents generating these data want to e.g. guarantee some desired level of differential privacy. This privacy mechanism consists in corrupting the information sent by the source with some (typically) Laplacian noise. Hence, it can be captured with a source having a Laplacian probability function. Moreover, Algorithm 1 does not determine the next state of the car but rather a probability function. This aspect can be used to guarantee some level of privacy for drivers using CRAWLING, making it possible to both privately share their policy (which can become a source for other vehicles equipped with CRAWLING) and determine the next direction by sampling from this probability.

### Making CRAWLING architecture openly available: implementation and validation set-up

**Figure 2:**
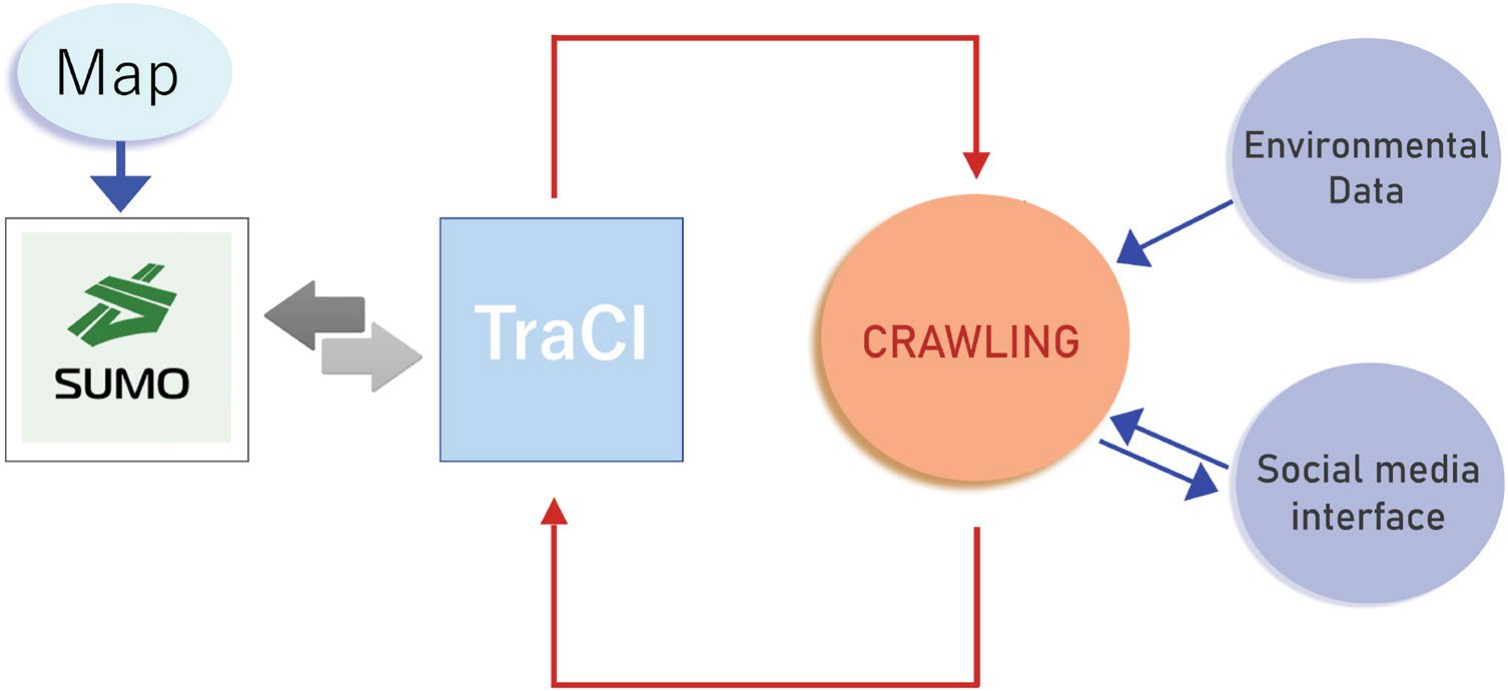
CRAWLING validation set-up. CRAWLING is implemented in Python and interacts with SUMO. This provides the back-end simulation from which CRAWLING gathers the relevant data through TraCI. The directions obtained via Algorithm 1 are fed back to the simulation environment via the interface TraCI. For example, in the validation scenarios described in Section “Scenarios description”, CRAWLING receives data such as parking availability or obstructed road links, which are used to compute directions to connected cars equipped with our service. CRAWLING also interfaces via a Social Interface module with information on social media (in Scenario 1.c, Twitter).

We now discuss the proposed implementation for the architecture of CRAWLING. The code can be found at https://tinyurl.com/wcwvcwaj. The key functional components of the validation set-up are illustrated in Fig. 2. As shown in such a figure, we validate our service via the microscopic traffic simulator SUMO^56^. This serves as the environment (in which real maps can be imported and traffic demand can be generated) with which CRAWLING interacts. Interactions with SUMO happen via the Python interface TraCI: this is used to both gather the environmental data from SUMO and to control the vehicles based on the directions provided by Algorithm 1. Moreover, the *Social Interface* module gives CRAWLING the possibility of interacting with certain social media (in our scenarios of Section “Scenarios description”, Twitter). In the set-up we propose, this is done by using the Python library *requests*, which can be used to interface with the Twitter API.

In the CRAWLING implementation, the discrete time steps are not uniform but are rather associated to the vehicle changing link (i.e., when the vehicle transitions state). This design solution removes the need for the cars to have a synchronized clock. Algorithm 1 is then implemented via a receding horizon strategy, thus allowing to handle possible changes in the environment. Specifically, every time (say, $t-1$) a given car equipped with CRAWLING transitions to a new link the algorithm:

- gathers the available data and sources (setting in Fig. 2);
- builds the reward based on new information that might have become available during the transition to the new link;
- computes the optimal plan of actions for the next *N* links according to Algorithm 1;
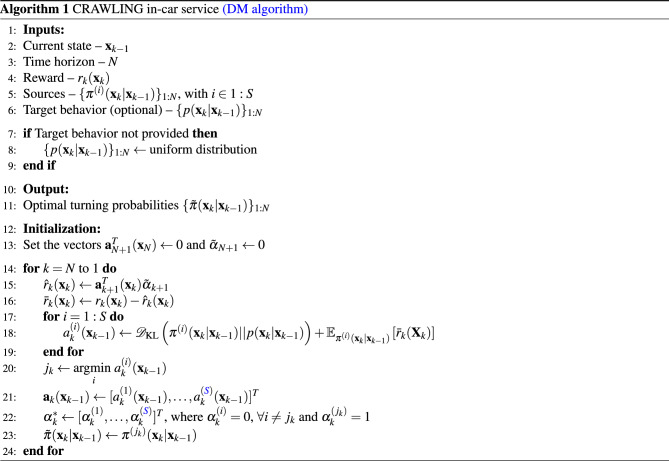
- determines the next direction by sampling from $\tilde{\pi }(\textbf{x}_{t}|\textbf{x}_{t-1})$. The car is then controlled through TraCI by setting the direction obtained from the sampling.

In our code (see our gitub repository for the details) connected cars are encoded by an *agent* Python class, which contains information about the user’s goal (i.e., the route/destination the driver vehicle would like to follow/attain). Algorithm 1 used by CRAWLING also has a Python class, which takes an agent as argument (i.e., a vehicle). This class regroups all the methods used for the algorithm, namely the computation of the KL divergence, a receding horizon control loop and a sampling mechanism. A simulation file is also provided to manage the key interfaces between the agent, CRAWLING and the simulator used to assess its performance (i.e, SUMO). Specifically, the simulation file includes functions for updating the state of the CRAWLING vehicle in the simulation, updating the reward and performing simulation steps through the SUMO-Python interface TraCI. The simulation files for Scenarios $1.\text {a}$, $1.\text {b}$ and 2 (see Section “Scenarios description”) also log results when the simulations are over, while the simulation file for Scenario $1.\text {c}$ (see Section “Scenarios description”) contains the necessary code to interface CRAWLING with social media, namely Twitter. A mechanism in CRAWLING is implemented that, when an obstruction is encountered by a connected car, accesses the twitter API and automatically shares information through a specific user’s account (one of the authors in this case). The content and date of tweets that have been shared in this way are also stored within the service so that other cars equipped with CRAWLING can parse the message and get access to the shared information. If the tweet parsed by our code has the hashtag ’#sumo_experiment’ it is considered for parsing. In this case, the function splits the string into individual words and searches for the word ’blocked’ (we leave the design of a more refined parser for future research) and for a road link identifier. Then, the date of the tweet is examined to ensure the tweet has an impact on the current trip: if the tweet was posted before the current date it is ignored. Finally, if all the conditions are met, the blocked road link is assigned a very negative reward as described in Section "Results". Note that, in the current version of the architecture, the tweets are stored within the service. This is due to recent changes to the Twitter API policy that now prevents the automated retrieval of a user’s tweet. However, on our github, we also provide a legacy version that allows to retrieve tweets, using the former version of the API. We decided to make openly available both versions to facilitate researchers wishing to build on CRAWLING: for example, for implementations in urban settings, we envision that CRAWLING could retrieve information shared by traffic authorities (through, e.g., social media). In this context, our architecture provides the necessary modular components to implement the service.

**Figure 3:**
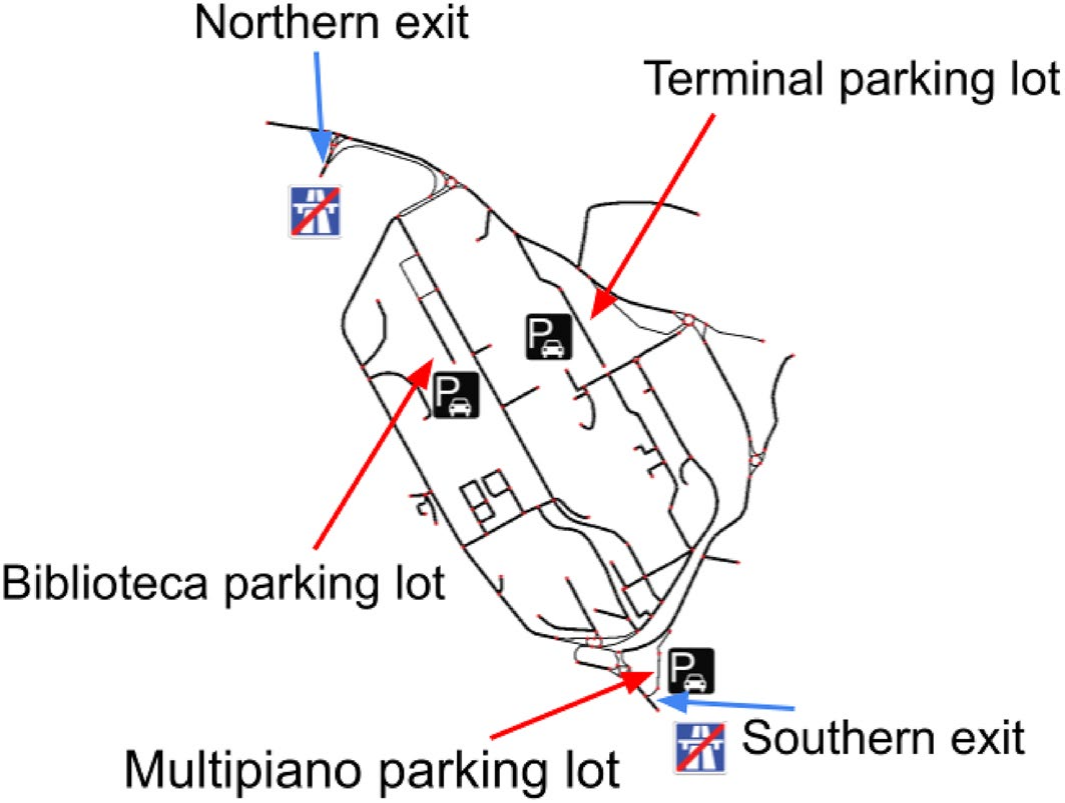
Campus road network. Highways and parking lots ate represented by their respective international symbols.

### Scenarios description

We now describe the scenarios (Scenario $1.\text {a}$, $1.\text {b}$, $1.\text {c}$ and Scenario 2 in what follows) that are used to validate CRAWLING. Specifically, Scenario 1 is centered around the management of a fleet of cars entering the University of Salerno campus and seeking to park. As we consider different conditions of interest within the university campus, Scenario 1 is split into three sub-scenarios, including an experiment aimed at verifying how CRAWLING’s social media interface would perform when spotting a closure. Scenario 2 takes place on a larger road network: the city center of Salerno.

**Scenario 1.a.** This scenario simulates a morning rush hour at the campus of the University of Salerno. The campus of the University of Salerno, shown in Fig. 3 is served by two highway exits, and has three parking lots (in our simulations, each parking lot can accommodate up to 50 cars). The highway exits and corresponding campus entrances will be referred to as southern and northern entrance and the parking lots as Multipiano, Terminal and Biblioteca parking lots (see Fig. 3 for the position of such parking lots on the campus map). In this scenario, a fleet of 150 cars (i.e., the maximum number of cars that can be accommodated in the simulation) arrives on campus before the teaching begins, with students and faculty staff searching for parking spaces. In particular, 100 cars arrive from the northern highway exit and seek to park on either Terminal or Biblioteca, while the 50 remaining cars arrive from the south and reach the Multipiano parking lot. The cars arrive on campus one by one at 15-second intervals, with the order of arrival being shuffled. That is, when each car arrives, its destination on campus and whether it is equipped with CRAWLING or not, are randomly selected. While cars arrive to campus, roadworks are being carried out on the main ramp leading from the northern highway exit to the campus, rendering traffic on the corresponding links (highlighted in blue in Fig. 7) difficult (the vehicles using such links have their speed restricted to less than one kilometer per hour). This ramp also corresponds to the preferred route of uncontrolled cars (that is, cars not equipped with CRAWLING) that seek to park in Terminal. In Section “Results” we use this scenario to benchmark the effectiveness of CRAWLING in properly managing the flow of cars. Simulations with varying amounts of controlled cars are performed to quantify performance.

**Figure 4:**
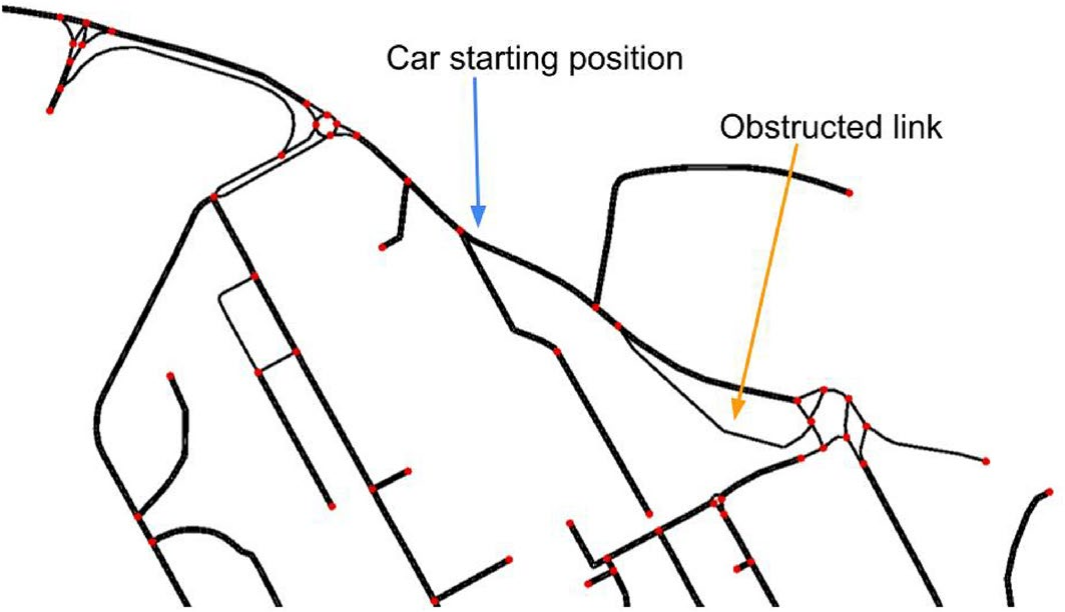
Setup for scenario $1.\text {b}$. The blue arrow shows the road link from where the cars are starting, after leaving Terminal, while the orange arrow shows the obstructed link. Colors online.

**Scenario 1.b.** This scenario also takes place at the University campus. However, it considers a different situation where some of the parking locations have been already saturated and incoming cars (e.g., late morning arrivals) need to park upon their arrival. Specifically, the more accessible Terminal and Biblioteca parking lots are full, as *early morning* users parked there upon arriving. Given this scenario, we consider a fleet of 50 cars that need to be parked within the University campus, after having attempted to park at Terminal, with the Multipiano (again can accommodate up to 50 cars) being the only parking location able accommodate new cars. Also, a road link on the path between the two parking lots is obstructed, see Fig. 4.

**Scenario 1.c.** In this scenario, we consider a fleet of 20 cars entering the campus from the northern highway exit, at 10-second intervals. The aim of the cars, equipped with CRAWLING, is to reach either the Terminal or Biblioteca parking. Within the scenario, the ramp leading directly from the highway exit to the parking lot is closed. Specifically, as the first car passes through the exit, the ramp is shut off completely and this information is shared through Twitter by CRAWLING. Other cars equipped with CRAWLING can parse the message and the service assigns a highly negative reward to the reportedly obstructed link so that incoming vehicles equipped with our service are re-routed to avoid the obstruction.

**Figure 5:**
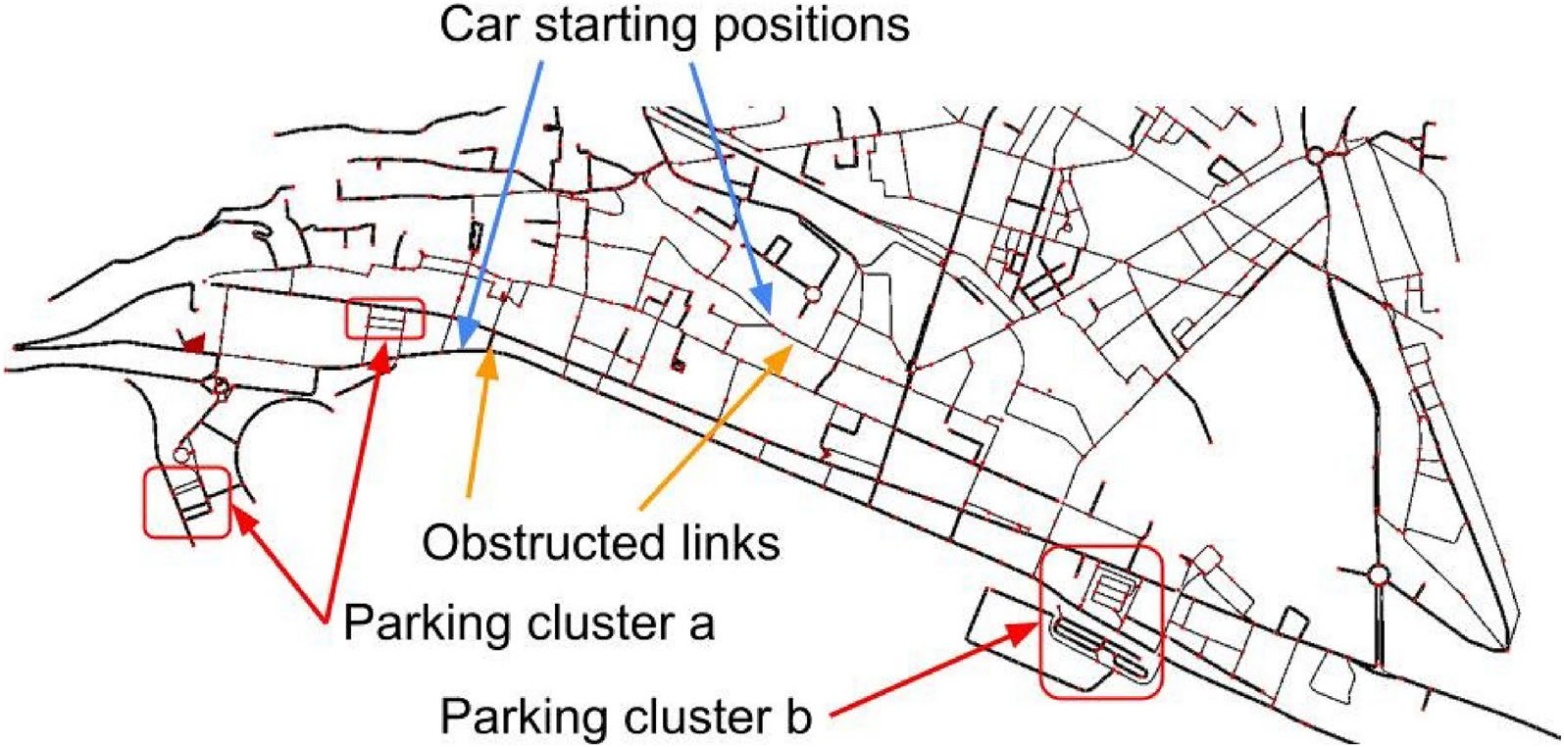
Salerno city center and setup for Scenario 2. Red arrows indicate parking lot clusters, blue arrows indicate starting positions for the cars and orange arrows indicate obstructions. Colors online.

**Scenario 2.** In Scenario 2 we deploy CRAWLING on a larger map: the city center of Salerno, emulating a situation (typical during e.g., the Christmas period when artistic lights^62^ attract a large number of tourists) where roads are congested and the area is also stressed by large parking demand. Parking lots are located around the harbors on the edge of the city. Cars enter from residential areas, and from high-capacity roads (coming from neighboring suburban areas). We generated a traffic demand to simulate cars entering in the city center in areas that are typically crowded. For concreteness, we create two points where 150 cars are generated and from these points cars seek to park in one of the city’s parking clusters. In total in the city center area there are 22 parking lots, each accommodating up to 25 cars with 21 parking lots distributed within the parking clusters and one located just outside of the city center. Additionally, we simulated (by obstructing links) a severe congestion in the very central part of the city. Within the scenario, we will verify that on this larger map CRAWLING allows the cars to bypass the obstructions, routing them to effectively find a suitable parking location. See Fig. 5 for details.

## Results

We now describe the results obtained with CRAWLING on the two scenarios of Section “Scenarios description”. To do so, we first empirically investigate the computational load incurred by CRAWLING (and discuss our design choices to make the algorithm suitable for routing applications) and then we describe the main settings of our simulation framework. The code can be accessed at https://tinyurl.com/wcwvcwaj. Note that, to replicate our simulations, a working installation of SUMO, TraCI, Tweepy and the main scientific computing Python libraries is required, along with a Twitter account with developer access.

### Computational performance

**Figure 6:**
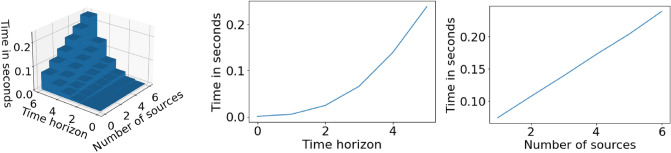
CRAWLING execution time as a function of available sources and time horizon. Leftward panel: computation time (in seconds) as a function of both time horizon and number of services. Middle panel: computation time (in seconds) as a function of the time horizon with 6 available sources. Rightward panel: computation time (in seconds) as a function of the amount of sources for a time horizon of 5 steps ahead.

To make CRAWLING suitable for in-car operation, we leverage the fact that cars being on a given link (i.e., state $\textbf{x}_{k-1}$) can only transition to an outgoing link (that is, $\textbf{x}_{k}$ can only be a link in the set of outgoing neighbors of $\textbf{x}_{k-1}$). This means that the support of the pfs $\pi (\textbf{x}_{k}|\textbf{x}_{k-1})$, $p(\textbf{x}_{k}|\textbf{x}_{k-1})$ and $\pi (\textbf{x}_{k}|\textbf{x}_{k-1})$ is restricted to the outgoing neighboring links of $\textbf{x}_{k-1}$ (see Table 1 for a reminder on notation). Hence, only the neighbors of $\textbf{x}_{k-1}$ need to be considered in the computations of Algorithm 1. Thus, in the algorithm implementation: (i) the set of outgoing neighbors associated to the current link of the car is determined; (ii) computations are subsequently performed only over the neighborhood (which depends on the time horizon) rather than over the full state space (which would amount to the full map). Using this approach, we empirically investigated if the service is fast enough for in-car applications. We did this by running CRAWLING on each link of the University Campus map in Fig. 3 and logging the average running time. On this map, each road link is connected, on average, to 7 other links. We repeated these simulations by varying, across them, both the amount of data sources available to the decision-maker (i.e., *S* was gradually increased between 1 and 6) and the time horizon (i.e., *N* was gradually increased between 0 and 5). The results are summarized in Fig. 6, leftward panel. The figure illustrates that the computation time is linear with respect to the number of sources (Fig. 6, rightward panel). This is due to the fact that Algorithm 1 iterates once per source at each time step. Also, the computation time is approximately exponential with respect to the time horizon (Fig. 6, middle panel). This aspect is essentially due to the exponential increase in the state space (even if reduced as described above) as the time horizon, *N*, increases. Interestingly, Fig. 6 shows that CRAWLING computation times are less than typical whole-link travel times^63,64^, thus confirming suitability of our implementation. These measurements were done on a standard desktop computer (Intel(R) Core(TM) i7-2600 CPU and 8GB of RAM) and we used Python 3.11.4 with SUMO 1.17 and TraCI 1.17.

### Simulation settings

We now report the settings that are used to implement the scenarios of Section “Scenarios description” and to obtain the results of Section "Results".

**Scenarios settings.** The parameters are set before running the simulation. In what follows we simply say that vehicles equipped with CRAWLING are *controlled* and vehicles without the service are *uncontrolled*. First, we created lists containing: (i) the starting points and target parking lots for uncontrolled vehicles; (ii) the target behavior for controlled vehicles. These lists are then shuffled to randomize the departing order of the vehicles in the simulation. For simulations involving both controlled and uncontrolled vehicles, the indexes of controlled and uncontrolled vehicles are randomly assigned. All this information is stored as .npy files. Namely: for Scenario 1, the files *agent.npy* and *foe.npy* contain this information. These files are generated before each simulation to avoid biased results. This can be done using the notebook *Simulation launcher.ipynb*. The relevant maps were imported from OpenStreetMap (https://www.openstreetmap.org/) using the netconvert software and cleaned up using netedit. A full tutorial for this procedure can be found in the SUMO online documentation. At the beginning of each simulation, SUMO loads the map, while TraCI adds each car from the. npy files to the simulation. The sources, stored as numpy arrays, are loaded when CRAWLING is launched. While SUMO runs the simulation, information including vehicle data and network conditions is obtained in TraCI and transmitted to CRAWLING, which builds the reward.

**Car settings.** All cars are implemented as TraCI agents. Controlled cars query CRAWLING for directions each time they transition to a new link, while uncontrolled cars are assigned a pre-defined trajectory at the beginning of the simulation (the trajectory is determined by the built-in SUMO routing function that determines the shortest path to the destination). Uncontrolled cars are set to automatically park at the end of their course if possible. On the other hand, controlled cars are assigned a parking space if they arrive to a non-full parking lot. If the parking lot is full, both controlled and uncontrolled cars are rerouted by assigning them a new, non-full parking lot (for uncontrolled cars this is done by assigning them a new destination in SUMO, while controlled agents are assigned a new target behavior).

**Figure 7:**
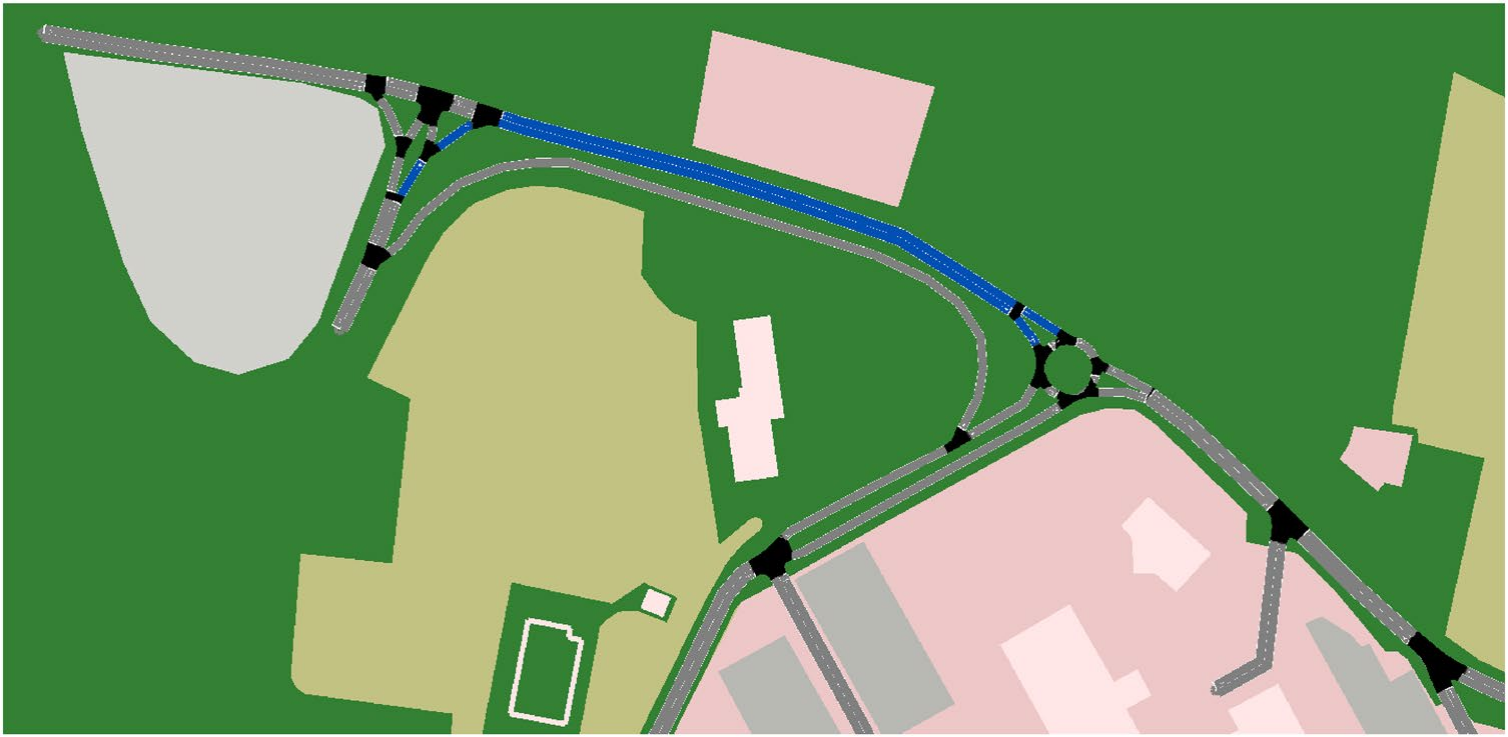
Northern section of the campus with obstructions highlighted in blue for Scenario 1. Map imported from OpenStreetMap in SUMO via its netconvert tool.

### Results

We now describe the simulation specifics and report the results.

**Reward and behaviors settings.** The reward encompasses environmental disruptions. In our use-case, this is computed from the state of the road links and is related to the availability of parking spaces. Specifically, at each time step, each link adjacent to a parking lot is assigned a reward of 100 if the parking lot has vacant spaces, and $-10$ if not. Road links on which traffic is perturbed are assigned an additional negative reward: (i) $-20$ if the traffic is heavily slowed down on the link. This has been simulated, as in Scenarios $1.\text {a}$, $1.\text {b}$ and 2, by enforcing a speed limit of less than one kilometer per hour on the perturbed road links; (ii) $-100$ if the road link is blocked altogether (as in Scenario $1.\text {c}$). Before running the simulations, a set of sources is compiled by using SUMO’s routing function: each source is designed to represent directions leading to a different point in the network. For the simulations of Scenario 1, we build 6 sources, using the highway exits, the parking lots and the north-western side of the campus as destinations. This ensures that the directory of behaviors available to the decision-maker provides a wide coverage of directions from each lane. For each road link, the probability functions $\pi ^{(i)}(\textbf{x}_k\mid \textbf{x}_{k-1})$ of the sources are built by assigning a high probability to the lane indicated by the routing while the other neighbor links have a small, uniform, probability of being selected. We also built a last source by merging behaviors of cars routed towards the Biblioteca and Terminal parking lot. This source can be thought of as emulating the traces from previous vehicles having navigated the campus. In the following, the target behavior of each connected car is selected among the sources. For Scenario 2, we build 22 sources having the parking lots as destinations (the probability functions for the sources, in. npy format, are provided on our github).

**Scenario 1.a specifics.** Our first set of simulations follows the first scenario outlined in Section “Scenarios description”. We implemented this scenario by simulating 150 cars arriving at the campus at 15-second intervals (with a simulation lasting approximately one hour and twenty minutes of simulated time) and seeking to park. All cars entering the campus via the Southern exit are directed towards the Multipiano parking lot. Uncontrolled cars entering through the Northern exit all seek to park in the Terminal parking lot. On the other hand, the target behavior of controlled cars entering through the Northern exit leads to either Biblioteca or Terminal (this can be interpreted as a target behavior being built from traces of previous CRAWLING cars having similar goals), highlighting the interest of obtaining directions through crowdsourcing stochastic policies. However, the road leading from the northern entrance to the roundabout immediately to its East is obstructed, causing traffic to significantly slow down (as described above, the speed limit on such road links is reduced to less than one kilometer per hour). As specified in Section “Scenarios description”, this obstruction occurs on the preferred route of uncontrolled cars directed towards Terminal, that is, uncontrolled cars within the 100 cars entering through the Northern highway exit. This is illustrated in Fig. 7, which captures a section of the full campus map. Such obstructions can originate from road work, or model a limited capacity of the road link related to e.g. severe climatic conditions. In the results shown next, we run different set of simulations with 0, 50, 100 and 150 controlled cars. This was done to evaluate the *penetration* rate of the service, showing its effectiveness as the number of cars equipped with CRAWLING increases.

**Figure 8:**
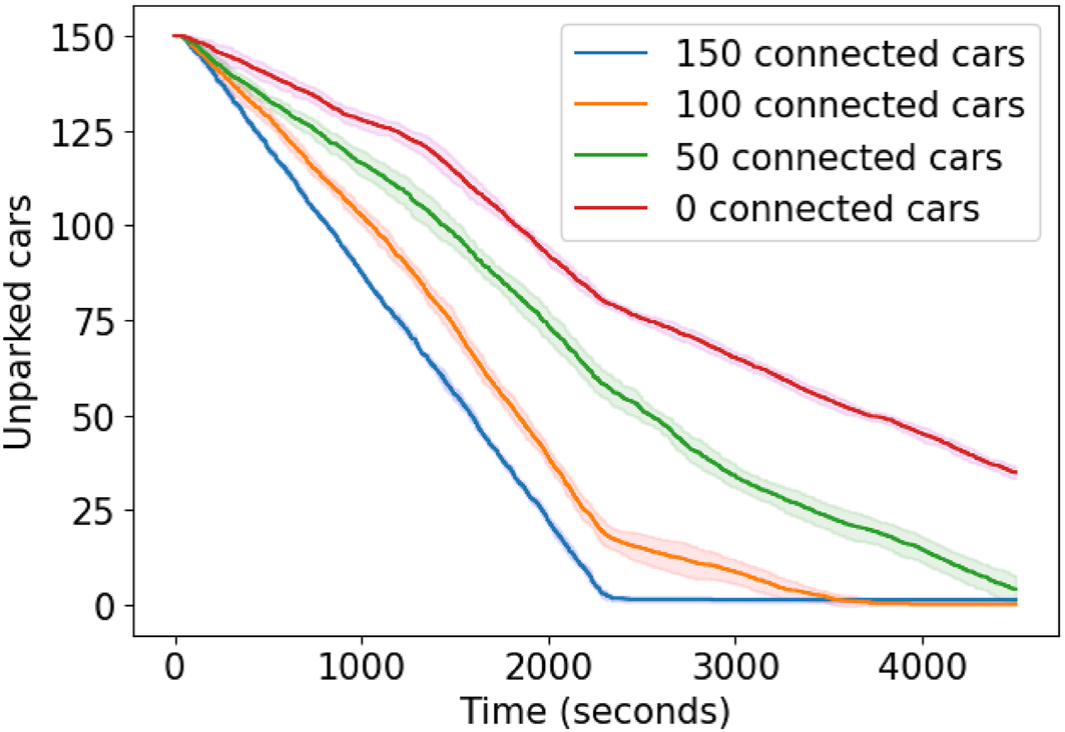
Confidence interval plot of the simulation results for Scenario 1.a. Solid lines represent the mean value, while the shaded area is the standard deviation. Fully controlled fleets manage to fully park within half of the simulation time, while fleets with less controlled cars take longer, failing to find parking spaces for every car before the simulation ends in the extreme case. Note that fleets with just one third of the cars using CRAWLING still significantly outperform the baseline of uncontrolled cars.

**Results for Scenario 1.a.** As shown in Fig. 8, the fully uncontrolled fleet (no vehicles equipped with CRAWLING) fails to park every agent in the allocated time. Indeed, all uncontrolled cars arriving from the northern highway exit are routed to the obstructed lanes. On the other hand, when the fleet is fully controlled (with all vehicles being equipped with our service), CRAWLING only needs approximately half the duration of the simulation to park every car. Namely, CRAWLING allows each controlled cars to adapt its route to avoid the obstruction, effectively orchestrating the use of the available parking spaces. Specifically, we observed that connected cars entering from the Northern exit were routed to the Biblioteca parking lot until it became full. When this happened, CRAWLING rerouted the remaining cars to the Terminal parking lot by a detour route avoiding the obstructed road links. Both controlled and uncontrolled cars entering from the southern highway exit reach the Multipiano parking lot, as it is the closest. To complement our analysis, we also recorded the average time spent by the average car on an obstructed link and the average time-to-parking over all simulations. These are shown in Table 2, together with the results from Welch’s t-test^65^, which we used to verify the statistical significance of our simulations. The test is aimed at checking whether two random samples originate from distributions having different means. In other words, a *p* value less than 0.05 would mean that a set of simulations is statistically insignificant and needs to be rejected. Specifically, we use the *p* value to compare the average parking time obtained in the five first simulations performed with a set of parameters with the five last simulations following the same parameters. Table 2 confirms the qualitative behaviors shown in Fig. 8. In particular, the increase in the number of cars equipped with CRAWLING decreases both the time spent on obstruction and the time-to-parking. This is due to the fact, with more cars being controlled via CRAWLING, these were able to coordinate to avoid the unfavorable road link, while being directed towards a parking with space with effective capacity to accommodate the incoming cars.

**Table 2:** Summary table for the numerical experiments. All times in seconds.

Scenario	Time (s) spent on obstruction	Average time-to-parking (s)	*p* value
Fully uncontrolled	754.2	2142.5	0.06
50 controlled	613.1	1999.0	0.63
100 controlled	280.2	1464.6	0.76
150 controlled	4.1	1186.2	0.46

**Scenario 1.b specifics.** We implemented this scenario (see Section “Scenarios description”) by generating, within the simulation, 50 cars at the exit of the road link leaving from Terminal (see Fig. 3) at 5-second intervals. Terminal and Biblioteca are both full, and the fleet’s target is Multipiano. Further, a link on the fleet’s preferred route (see Fig. 4) is obstructed. We performed two sets of simulations: one with a fleet with all cars equipped with CRAWLING and the other with no cars equipped with CRAWLING.

**Figure 9:**
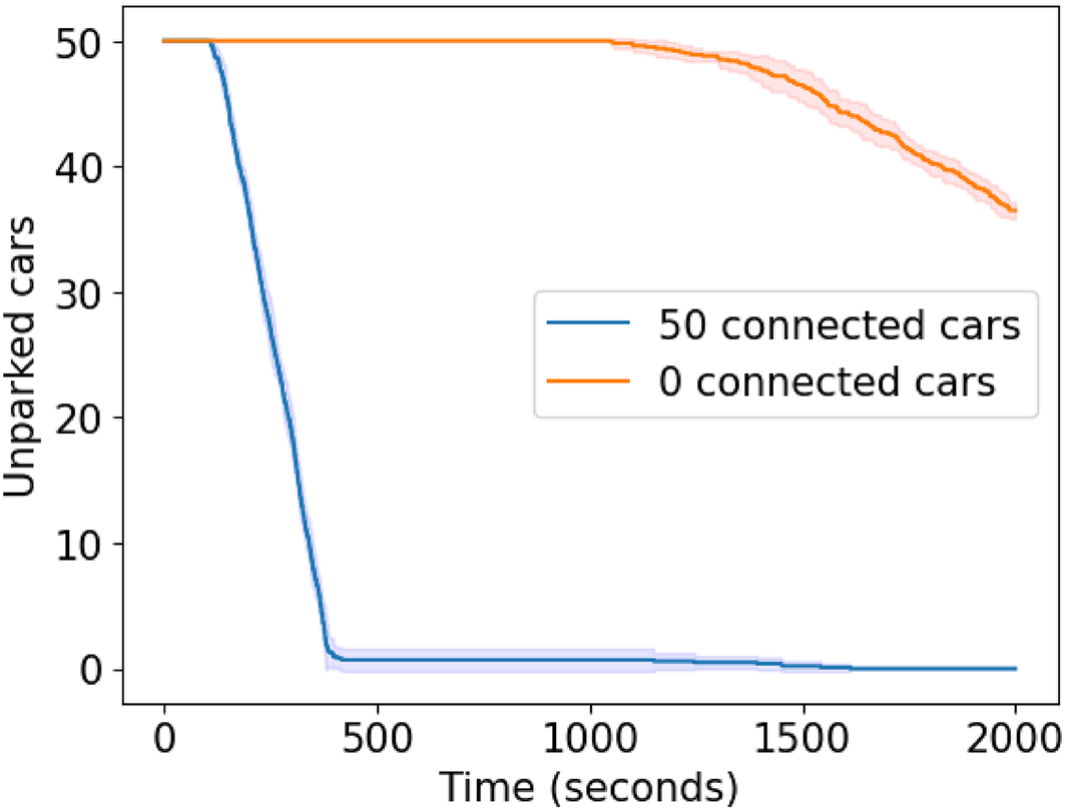
Confidence interval plot of the simulation results for Scenario 1.b. Solid lines represent the mean value, while the shaded area is the standard deviation. The fleet equipped with CRAWLING manages to avoid the obstruction. When cars do not have the service, they spend high amounts of time navigating the obstructed link.

**Results for Scenario 1.b.** As shown in Fig. 9, when the cars are equipped with CRAWLING, these all find parking within the simulation time. This does not happen when cars are not equipped with our service. The observed behavior is due to cars equipped with CRAWLING being able to circumvent the obstruction via an alternative itinerary. Specifically, after the detour, these cars return to the preferred route, arriving to their destination and parking faster than their counterparts.

**Scenario 1.c specifics.** For Scenario $1.\text {c}$, half of the cars seek to park in Terminal, and half in Biblioteca. However, as the first car exits the highway, the exit ramp it is driving on is blocked. If a car is on this link, a tweet is automatically sent out by CRAWLING through the Twitter API. Specifically, *North highway ramp blocked* $\#$*sumo_experiment* is automatically posted by CRAWLING (in this scenario via one of the authors twitter account). The content of the tweet is also stored by CRAWLING so that other cars equipped with our service can parse it (see Section "Making CRAWLING architecture openly available: implementation and validation set-up"). At each time step, if the parsing mechanism detects an obstruction, CRAWLING assigns a very negative (i.e., $-100$) reward to the road link where the disrupting event is signalled.

**Figure 10:**
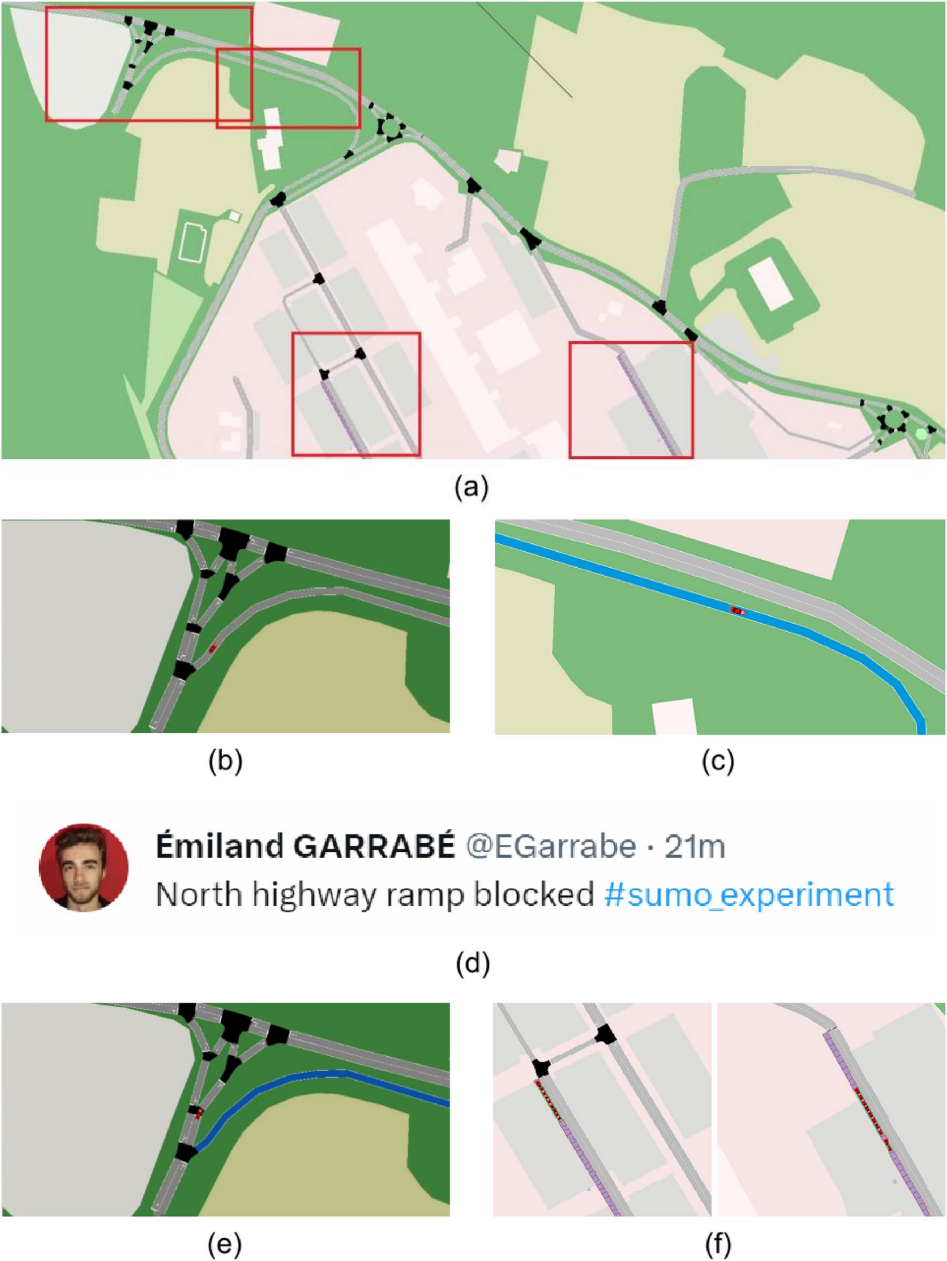
Snapshots from the simulation for Scenario 2. Panel (**a**): areas of the campus interested by the use-case. The red rectangles denote the areas in panels (**b**),(**c**) and (**f**). Panel (**b**): first car taking the fastest route from the Northern entrance when there is no obstruction. Panel (**c**): the route is blocked, causing the first car to slowly proceed through the obstruction. Panel (**d**): the information about the obstruction is shared via a tweet. Panel (**e**): CRAWLING adapts to the disruption and the following cars use an alternate route. Panel (**f**): the cars reach their destinations after bypassing the obstruction.

**Results for Scenario 1.c.** The results are summarized in Fig. 10. Such a figure illustrates, in panel (a), the key campus areas for the scenario. At the beginning of the scenario, no road obstruction is detected and indeed, as shown in panel (b), the first car entering in the simulation normally transitions through the Northern exit, following the shortest path through the ramp. As the car passes the ramp, the road obstruction occurs (the obstructed link is highlighted in blue in panel (c)) and the obstruction is reported via a tweet (in this experiment, posted on one of the author’s account, see panel (d)). Panel (d) showcases the corresponding tweet that was issued upon detection of the road blocking. Then, panel (e) shows the result of CRAWLING adapting to the new information. Indeed, the route for the following cars is re-computed by our service to prevent following cars from entering in the obstructed road link. This is a result of CRAWLING detecting the reward change due to the tweet. An interesting question is to determine a measure of trust for the tweets to be used by CRAWLING. We leave this topic for our future research. At the end of the simulation—panel (f)—rerouted cars achieve their goal of reaching parking spaces. A video of the corresponding simulation is also available at https://tinyurl.com/yc7fy2j2.

**Scenario 2 specifics.** Cars enter the simulation at 5-second intervals, with the car’s departing time, place and (for mixed simulations) whether they are equipped with CRAWLING or not being randomly shuffled as in Scenario $1.\text {a}$. The cars generated on the western starting position seek to reach parking cluster a, while the cars generated to the east seek to reach cluster b (see Fig. 5 for details). The obstructed road links are also shown in Fig. 5, and on these links the maximum speed is restricted to less than one kilometer per hour. Each of these obstructions is on the preferred route of the cars. We performed simulations with fleets containing 0, 100, 200 and 300 cars equipped with CRAWLING to benchmark the service and investigate its penetration rate on this larger scenario.

**Figure 11:**
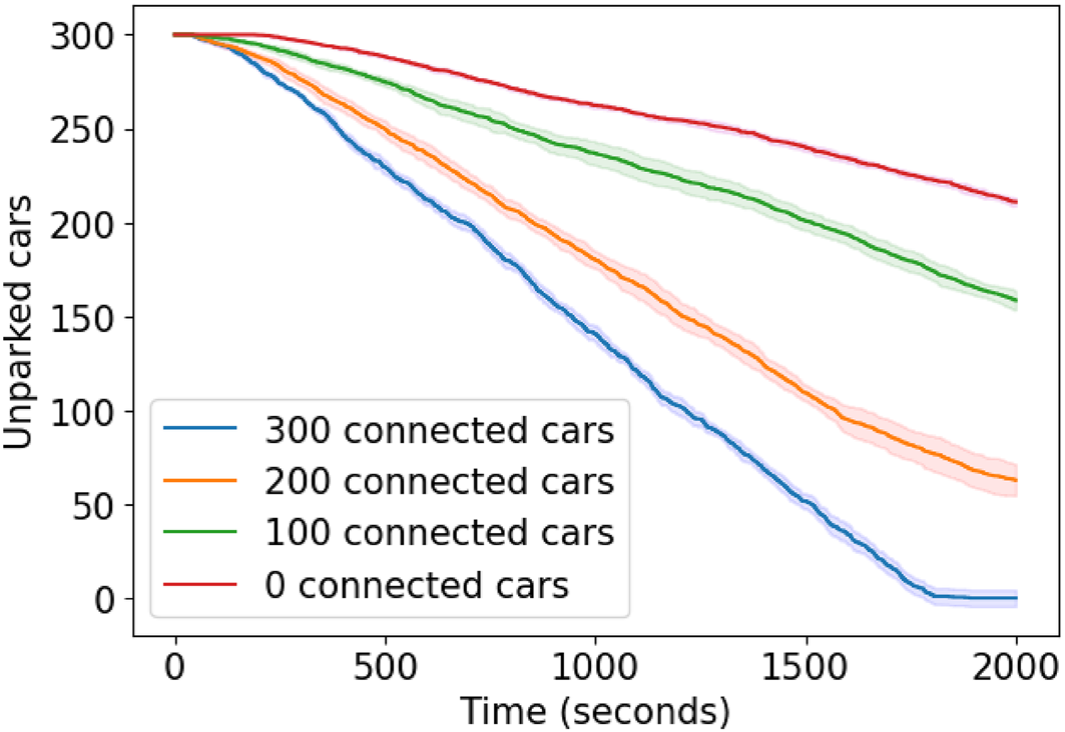
Confidence interval plot of the results for Scenario 2. Solid lines represent the mean value, while the shaded area is the standard deviation. The fully connected fleet reaches the target parking lots significantly faster than its non-connected counterpart. The legend shows, for each set of simulations, how many cars within the fleet are equipped with CRAWLING.

**Results for Scenario 2.** Again, when no vehicle is equipped with CRAWLING, the fleet fails to park in the allocated time. Instead, cars equipped with CRAWLING adapt their route to avoid the obstruction and effectively find parking. Simulations showed that the fleet with all cars equipped with CRAWLING achieves its goal significantly faster than the fleet when no cars have our service (see Fig. 11). Similarly to Scenario $1.\text {a}$, CRAWLING is also able to redirect cars that were initially driving towards a parking lot that became full, as this change in conditions is captured by the reward and informs the in-car system’s online decision-making. Interestingly, the results illustrated in the figure are consistent with these obtained within the smaller Scenario 1.a, in turn showing the suitability of CRAWLING to handle such larger-scale scenarios.

## Discussion and concluding remarks

We presented the principled design of CRAWLING, an in-car service for smart parking, for which we make our code available. The service leverages a recent data-driven optimal control algorithm, where the cost function has a Kullback-Leibler divergence component, ensuring that the car tracks a certain target behavior, i.e. a route that the user would like to take. A reward signal is also incorporated into the cost, compiling additional information that might be collected through a network of urban sensors and on social media. A key feature of our service is that it determines turning probabilities for cars. Hence, the *policy* that is computed by the service is stochastic and it allows to both embed in the framework the possibility that drivers do not follow the indications provided by CRAWLING and to guarantee some desired level of e.g., (differential) privacy for users participating to the service. After describing the key functional components of CRAWLING, we proposed a stand-alone, general-purpose modular architecture of our service. We introduced the components of this architecture and our simulation framework, which we used to investigate the service's effectiveness on different Smart Cities scenarios, leveraging SUMO for microscopic traffic simulations. With our first scenario (morning rush) we investigated what happens as the number of vehicles equipped with CRAWLING increases, empirically finding that parking times can be considerably improved even when only $30\%$ of the vehicles were controlled by our service. These simulations were also performed with a limited amount of sources, providing encouraging insights into the system’s ability to efficiently make decisions based on a limited set of available services. These results were confirmed in our second and fourth scenarios, with CRAWLING significantly speeding up the fleet’s search of parking spaces in different operating conditions, thus illustrating that CRAWLING is suitable for operation on various networks and under different conditions. With our third validation scenario, we investigated the effectiveness of CRAWLING to adapt to environmental changes via social media: simulations once again confirmed the benefits of equipping cars with our service.

In a broader context, a number of considerations can be drawn from our findings. While for concreteness we tailored CRAWLING towards parking management, its approach can be exported to other routing applications. We see our findings as a first step to implement an *internet of skills* for connected cars. In this framework, we envisage cars to collaboratively exchange learned skills so as to create a collective intelligence enabling cars to augment each others’ abilities through crowdsourcing. We also note that some ventures have already started delivering services based on data collaboratively shared by their users, see for example the Waze application^66^. Interestingly, similar mechanisms based on crowdsourcing can be found in neuroscience—see, for example the recent *thousand brains* theory^67^, which postulates that the neocortex in our brain does not compute brand new *behavioral models* but it patches simpler models from the cortical columns. Finally, in the context of reinforcement learning, a mechanism related to CRAWLING has been recently proposed to orchestrate the use of learning-based and model-based policies to tackle certain control tasks. Interestingly, it has been shown that mechanisms orchestrating these heterogeneous policies can be designed so that they can tutor each other and this, in turn, improves learning efficiency and performance^68–70^. Inspired by these studies, possible future directions of our research might involve both embedding tutoring mechanisms in CRAWLING and exploring the links between the approach we presented and certain recent neuroscience theories. Another point of interest is to embed into our architecture a recently developed decision-making engine^50^ that allows agents to merge behaviors and that has been only tested on a simplified scenario, without a social media component. We therefore plan to assess the performance of CRAWLING when different decision-making engines are used. We will also embed strategies to filter social media information for their effective use in CRAWLING. Finally, we aim at investigating whether CRAWLING can represent an effective mechanism to fairly regulate possible competitive behaviors between vehicles equipped with our service and legacy, manually-driven, vehicles when these compete for the same, limited, resource.

## Data Availability

The code, and the corresponding detailed instructions, needed to replicate the simulations can be found at: https://tinyurl.com/wcwvcwaj.
